# Large-scale regulatory and signaling network assembly through linked open data

**DOI:** 10.1093/database/baaa113

**Published:** 2021-01-18

**Authors:** M Lefebvre, A Gaignard, M Folschette, J Bourdon, C Guziolowski

**Affiliations:** UMR 1332 Biologie du Fruit et Pathologie, INRAE, Univ. Bordeaux, 72 Avenue Edouard Bourlaux, CS20032, 33882, Villenave d’Ornon cedex, France; Université de Nantes, CNRS, INSERM, l’Institut du Thorax F-44000, Nantes, France; Univ. Lille, CNRS, Centrale Lille, UMR 9189 - CRIStAL - Centre de Recherche en Informatique Signal et Automatique de Lille, F-59000 Lille, France; Université de Nantes, Centrale Nantes, CNRS, UMR 6004 - LS2N - Laboratoire des Sciences du Numérique de Nantes, F-44000 Nantes, France; Université de Nantes, Centrale Nantes, CNRS, UMR 6004 - LS2N - Laboratoire des Sciences du Numérique de Nantes, F-44000 Nantes, France

## Abstract

Huge efforts are currently underway to address the organization of biological knowledge through linked open databases. These databases can be automatically queried to reconstruct regulatory and signaling networks. However, assembling networks implies manual operations due to source-specific identification of biological entities and relationships, multiple life-science databases with redundant information and the difficulty of recovering logical flows in biological pathways. We propose a framework based on Semantic Web technologies to automate the reconstruction of large-scale regulatory and signaling networks in the context of tumor cells modeling and drug screening. The proposed tool is pyBRAvo (python Biological netwoRk Assembly), and here we have applied it to a dataset of 910 gene expression measurements issued from liver cancer patients. The tool is publicly available at https://github.com/pyBRAvo/pyBRAvo.

## Introduction

Systems biology is a research area aimed at obtaining a better understanding of biological interactions at several levels. The associated activities require combining multiple databases to integrate information, and computational modeling (1) addresses the complexity of this integration process. Graph-based modeling approaches are particularly suitable for elucidating biological systems because they structure information in an organized (possibly causal) way to represent the relation between complex components such as genes, proteins and protein complexes. For instance, graph structures represent inhibition of activation in gene regulatory networks (GRNs) or protein stimulation in signaling networks (SNs). These networks can be easily transferred to computational models, which can perform complex combinatorial analyses or simulations to extract network properties or predict system states. Several systems biology approaches use computational models built upon biological networks to understand human diseases. For example, researchers have previously undertaken the construction and kinetic modeling of a Parkinson’s disease interaction map (2), while others have combined the cross-talk of multiple T-cell receptor pathways to understand immune response (3).

To assemble such graphs, multiple data or knowledge bases must be integrated. This integration effort is currently facilitated through several initiatives aimed at better organization of biological knowledge and its open availability, such as Reactome (4), Kyoto Encyclopedia of Genes and Genomes (KEGG) (5), PathwayCommons (PC) (6), WikiPathways (7), Pathway Interaction Database-NCI (8), Consensus PathDB (9), OmniPath (10) and hiPathDB (11), among others. These databases can be automatically queried to reconstruct a large variety of biological networks such as GRNs or SNs.

Some of these databases, such as KEGG, WikiPathways, PC ChiBE (12) and Reactome, provide an explicit representation and a user-oriented visualization to better understand biological phenomena. However, they do not include query mechanisms to automate the reconstruction of possibly complete (across multiple pathways) upstream subgraphs from a list of genes of interest. For other databases, specific tools have been developed to automate network reconstruction from a list of input genes. These tools include CyPath2 (13), PCViz (https://www.pathwaycommons.org/pcviz/), PyPath (10) and ReactomeFIViz (14), all of which produce graphs representing biological networks. However, due to their modeling choices, derived computational models often fail in representing causal biological flows. For example, the CyPath2, PCViz, ReactomeFIViz and PyPath tools represent protein complexes as a set of interacting entity members (cycles) without including the complex itself as a node in the graph. In contrast, KEGG, WikiPathways, ChiBE and Reactome visualization tools keep protein complexes in the visualization as nodes. The first modeling choice may strongly influence computational modeling because cycles can generate oscillations in simulations. These artificial oscillations do not represent a biological reality.

Single-source tools for reconstruction of GRNs and SNs such as ReactomeFIViz and KEGGscape (18) do not enable tackling the challenges arising from the integration of biological data from multiple sources. PC and OmniPath are two large-scale initiatives in that direction: they integrate several existing data sources—PC covers 24 resources and OmniPath covers 34 resources—thus allowing to combine multiple knowledge bases. However, scientists face a massive amount of query results when querying resources, and they consequently need efficient and scalable algorithms to exploit their richness.

We identified the following issues that need to be addressed to enable better modeling of biological systems: (i) combining multiple data sources, possibly at multiple scales; (ii) automating the exhaustive reconstruction of multi-source multi-pathway networks and (iii) leveraging biological semantic models to represent causal biological flows. In this paper, we propose pyBRAvo as a computational framework based on Semantic Web technologies (BioPAX ontology) and multiple public data source (PC) to automate the reconstruction of human multi-source and multi-pathway GRNs and SNs.

## Material and methods

### PathwayCommons

PC  (6) is a large-scale initiative aimed at integrating biological pathway data collected and curated from multiple data sources and making the information accessible. At the time we evaluated our tool, PC version 11 encompassed 22 data sources, 9500 pathways, 3 million interactions and 1.5 million entities. In the updated version 12, PC integrates 22 data sources and provides information on more than 11,500 pathways, 2.4 million interactions and 1.2 million entities. Some of the data sources focus on pathway information such as SMPdb, Reactome, KEGG or WikiPathways, while other sources focus on biological interactions such as BioGRID or IntAct. PC relies on Linked Data (15) principles to make this massive pathway knowledge interpretable from both human and computational points of view. Linked Data provides standards to represent and link resources as public knowledge graphs on the web, and resource description framework (RDF) (16) is the W3C standard used to represent these knowledge graphs. It is particularly suitable for representing biological knowledge because it allows the representation of directed labeled graphs. In addition, these resources as well as their relations can be strongly typed with controlled vocabularies or more formal ontologies. In particular, PC leverages the BioPAX ontology (17) to provide a standard and uniform view of these multiple and diverse databases. Finally, thanks to the support of Semantic Web standards and technologies, especially the SPARQL query language, this massive biological pathway knowledge graph can be queried on live up-to-date biological data to mine and retrieve specific graph patterns.

### Gene regulatory and SNs reconstruction

#### Network reconstruction algorithm

**Figure 1.**
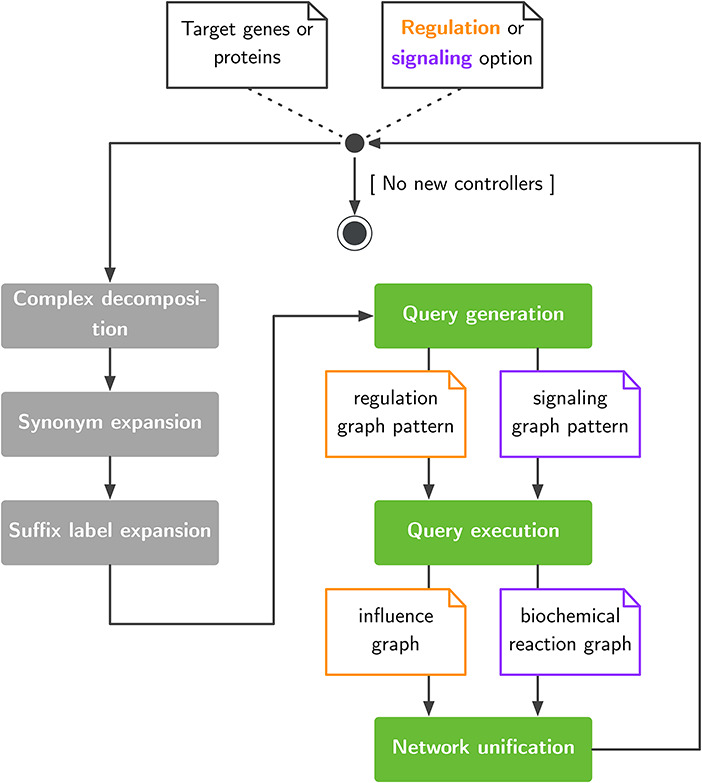
Exploration of large pathway databases to assemble gene regulatory and signaling networks.

With pyBRAvo, we propose a method to automatically assemble GRNs and SNs based on query rewriting. pyBRAvo leverages publicly available datasets through the PC initiative. Figure 1 presents the network reconstruction algorithm. pyBRAvo first takes a list of genes or protein names as input. This list is then augmented with three optional text-based processings (gray boxes) aimed at decomposing protein complexes, inserting synonyms and inserting suffixes commonly found in PC. These processings are described in the ‘Query expansion and network unification’ section. Then, the query generation step produces two pattern-matching expressions (see Supplementary Methods A.2) according to the specific kind of network to be reconstructed (i.e. GRNs or SNs). The generated query is executed on the remote PC data source, and results are recursively explored until no new controllers can be found or the maximum exploration depth has been reached. Users obtain their results in the form of a formal graph, which can be either an influence graph or an hyper-graph (see Supplementary Methods A.3).

#### Query expansion and network unification

To match the maximum number of entities during the network reconstruction process, we propose the following optional pre- and post-processings, as shown in Figure 1.

##### Complex decomposition.

Protein complexes can be identified in BioPAX knowledge bases either through their semantic type (bp:Complex) or by a label concatenating several names, separated with special characters. For performance concerns, we favored a syntactical approach that consists of splitting the label into several names and adding these names to the list of biological entities to be explored in following iterations.

##### Label expansion.

Although BioPAX allows modeling several forms of biological entities, some databases express specific forms through a prefix or a suffix in entity labels, such as *expression of* or *mutant form*. To maximize entity retrieval, we dynamically expand the regulation and signaling queries with a set of predefined prefixes and suffixes when reconstructing the interaction network. These prefixes and suffixes are removed when entities are added to the regulation or SN.

##### Synonym expansion.

To enhance the matching of genes or protein names, we account for common synonyms as registered in the NCBI Gene Info database (Homo_sapiens.gene_info). For each biological entity name retrieved through PC, we extend the regulation or signaling queries with their synonyms for further exploration.

##### Network unification.

Finally, we reuse the NCBI Gene Info database to merge synonym graph nodes into nodes identified with their common name. This computation (of merging synonyms) is done for each analysis. Thus, two graphs are always proposed: one that is unified and another that is not.

### Experimental datasets

Two datasets are used in this work as the basis of use-cases to illustrate the application of pyBRAvo. Both datasets pertain to the study of human hepatocellular carcinoma (HCC).

#### EMT signature-based dataset of 910 genes

A gene expression profile based on the differential expression (DE) of 16 283 genes in human liver cells was obtained from the LIHC-US project (Liver HCC—TCGA, US, Release 21. https://dcc.icgc.org/releases/release_21/Projects/LIHC-US) of the International Cancer Genome Consortium (ICGC) database (18). The RNAseq expression of these genes was measured across a population of 294 patients with liver cancer. The DE of genes was obtained by comparing patients with liver cancer at an invasive stage with patients whose cancer was at an early and non-invasive stage. Similar to a previously used approach (19), this comparison was based on the detection of epithelial–mesenchymal transition (EMT), the process by which cells acquire invasive and migratory abilities. The output of this DE analysis is a list of 910 genes that were notably over-expressed (fold-change > 2) or under-expressed (fold-change <−0.5) under the constraint *P* < 10^−5^. These 910 genes are used as input for our software in the ‘Gene regulatory network reconstruction’ and ‘Signaling network reconstruction’ sections.

Metabolic model-based dataset of 39 genes. In (20), pyBRAvo was used to feed a probabilistic model aimed at predicting the impact of drugs on cell growth rate in the context of HCC. More precisely, in this use-case, we used the iLivercancer1715 model proposed by (21). This metabolic model consists of 4663 metabolic reactions and 5735 metabolites, together with an objective function that describes the biomass growth of the HCC tumor cell. Since the model is reconstructed based on the human metabolic reaction model, deriving the list of 2881 genes with a direct effect on the metabolic reactions of HCC is straightforward. Notice that a single reaction deletion analysis can be used to filter the obtained list to the genes having an effect on the objective function of the metabolic model, similar to a flux balance analysis. This filtered list, containing **39 genes**, serves as the list of target genes in an upstream gene regulation network reconstruction by pyBRAvo in the ‘Gene regulatory networks for drug screening applications’ section.

### Evaluation metric computation: coverage

To illustrate the construction of SNs (see the ‘Signaling network reconstruction’ section), we compared pyBRAvo (see the ‘Query expansion and network unification’ section) with the algorithm proposed using PyPath functionalities (see the ‘Signaling network reconstruction’ section). Both tools retrieve graphs from a list of input genes. The coverage metric used for this study measures the number of input genes belonging to the output graph. It is computed based on the following formula:

$$\begin{equation*} S = |{\rm ON}| \div |{\rm IG}| \times 100 \end{equation*}$$

where ON is the set of input genes belonging to the output graph and IG is the set of input genes, ON ⊆ IG. This metric represents how well the knowledge of the database, extracted with the specific tool (pyBRAvo or PyPath), covers the initial gene dataset. The coverage was used to interpret our results when reconstructing SNs and GRNs.

## Results

We applied pyBRAvo to reconstruct two different types of networks using the same gene expression profile. In the first one (‘Gene regulatory network reconstruction’ section), we built a regulatory network and modeled it using Iggy, a tool based on Answer Set Programming that performs a *sign-consistency* analysis comparing biological networks with experimental observations (22). In the second one (‘Signaling network reconstruction’ section), we used pyBRAvo to reconstruct an SN and compared the obtained results using PyPath, a Python package designed to query the OmniPath database (version of October 2019) to work with molecular network interpretations from this database. Finally, we show in the ‘Gene regulatory networks for drug screening applications’ section how pyBRAvo can be used in a drug-screening application to enrich potential target genes and associated candidate drugs.

### GNN reconstruction

We used pyBRAvo to query the PC database (version 11), excluding both miRTarBase and MSigDB sub-databases, to generate a regulatory network that explains the upstream events of the 910 genes selected based on the EMT signature (see the ‘Experimental datasets’ section). The miRTarBase database was excluded because it contains negative interactions due to miRNA entities, for which we had no experimental observations. MSigDB was excluded because of the absence of signs/roles in the effect of all its interactions; MSigDB interactions are obtained via computational predictions on the gene binding sites. The reconstruction was limited to a recursion depth of 10 levels. This graph was obtained using the Python command line script of pyBRAvo (Command: python pyBRAvo.py -reg -md 10 -sy -su -co -f input.csv -excl mirtarbase msigdb); this call used the search of synonyms, complex decomposition and label expansion options explained in the ‘Query expansion and network unification’ section. The search took 25 minutes and 11 seconds of computation time (On a standard laptop computer with an Intel Core i7-6600U CPU of 4 × 2.60GHz, 16Gb memory and running Ubuntu 18.04.) when the private SPARQL endpoint was used. Computing the same pyBRAvo call using the PC SPARQL endpoint took 98 minutes and 9 seconds.

We obtained a directed and partially signed graph composed of 1678 nodes and 4425 edges, among which 3719 are signed (as *activation*, *inhibition*) and 599 are labeled by pyBRAvo as PART_OF to denote the oriented relation between a protein and the protein complex it belongs to. Among the 1678 nodes, 330 can be identified as protein complexes, and 27 of them are protein complexes of small molecules; the other 1321 nodes can be identified as proteins or genes. The edges of this graph represent transcriptional regulation interactions and complex formation interactions. From the 910 queried genes, pyBRAvo recovered the upstream events for 691 (76% of coverage) of them. The reason that not all 910 original genes were found is that not all genes are documented in the queried resources of PC as being regulated by transcription factors. Independently from this study, we used the same list of 910 genes to query the KEGG database for upstream (transcriptional) regulators and we obtained only 63 genes (6% coverage) that were upstream regulated.

#### Provenance

**Figure 2.**
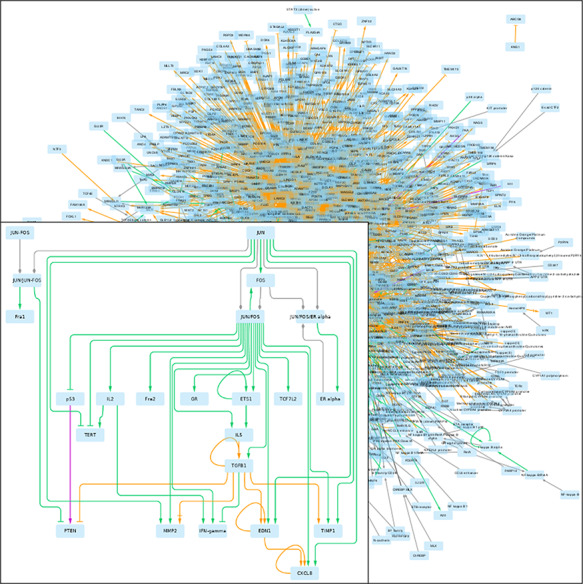
GRN returned by pyBRAvo. The color code of the edges gives their provenance: orange for CTD, purple for Panther, green for PID, red for Reactome and gray for the expansion of protein complexes. The edge tips depend on the interactions type: triangle for an activation, bar for an inhibition, arrow with bar for an unknown interaction type and diamond for a complex composition. This graphical export was made with Cytoscape 3.7 (13), following the instructions of Supplementary Methods G.

In this case study, we were interested in understanding how different databases provide information that enables the construction of a graph explaining the EMT signature genes. In Figure 2, we show a graph that was retrieved; its edges are colored according to the source database. The Cytoscape session of this figure is available online (https://github.com/pyBRAvo/pyBRAvo/blob/master/cytoscape-vis/regulation_paper_sept.cys). In Table 1, we show the number of different edges retrieved per database. Since Pathways Commons information remains highly heterogeneous, each database uses its own vocabulary to represent the knowledge. Therefore, the options of *complex decomposition*, *synonym expansion* and *label expansion* provided by pyBRAvo are useful to obtain a more complete and less disconnected graph. Indeed, without these options, the graph has only 172 nodes and is made of five disconnected cliques.

**Table 1.** Distribution of the provenance of the 4425 transcriptional regulation edges returned by pyBRAvo

Database name	Edges	Signed edges
CTD	3136	3052
PID	646	646
Panther	42	19
Reactome	2	2
pyBRAvo (PART_OF)	599	

The first column gives the name of each database that returned edges, where PART_OF stands for pyBRAvo’s specific result: edges representing the association of proteins with the protein complexes they belong to. The second column gives the total number of edges found in the database, which can be of signed type (*activation* or *inhibition*) or unsigned type. The third column gives the number of signed edges among them.

#### Feeding computational modeling tools for Systems Biology users

What we understand as a computational model is a mathematical framework that uses either a computational simulator or a solver to discover underlying properties of a (biological) graph, such as a computational prediction of unobserved species behavior. In a computational modeling tool, mathematical equations, logical rules or constraints are involved. One of the motivations in conceiving pyBRAvo was to ease the automatic transfer from knowledge databases toward such computational models.

To illustrate this transfer, we built a discrete mathematical model integrating the pyBRAvo regulatory network without the 106 unsigned edges, obtained from Pathways Commons, and the up- and down-regulation DE measurements of the 910 genes obtained from ICGC. This mathematical model follows the *sign-consistency* approach implemented in the Iggy framework (23), which uses *clasp*, a conflict-driven Answer Set solver used in the study of NP-hard search problems (24). Iggy tests the consistency between the network logic (signs and directionality) and the DE information of the genes (up/down shifts of regulation). Interactions labeled as activations and inhibitions were modeled by Iggy using plus and minus signs, respectively. Interactions labeled as *PART_OF* were modeled by Iggy using a plus sign, and we considered protein complex formations as positive influences. The model had to be minimally corrected in 148 interactions before being consistent with the experimental observed up/down shifts. Afterwards, we obtained 82 predictions in unmeasured network components (see Supplementary Table S3). We validated these model predictions by comparing them with the original DE data of the genes that were not used for the graph reconstruction or as experimental observations. Here, we consider that a gene with positively (resp. negatively) differentially expression should be predicted positively (resp. negatively). Among the 82 predicted genes, 63 were found in the 15 373 genes from the experimental DE data. Of these 63 genes, 29 had a prediction matching the experimental data and 34 had a converse prediction. It is beyond the scope of this paper to analyze these computational prediction results. However, we deemed it important to present a concrete example of how pyBRAvo processing of information can provide a bridge from the PC regulatory knowledge to concrete modeling frameworks such as Iggy. In Supplementary Table S4, we give details for this case study concerning the evolution of the graph retrieved using the same pyBRAvo options for 2 and 10 levels of depth.

### SN reconstruction

In this section, we illustrate how SNs, in the forms of *influence graphs*, are obtained with pyBRAvo (see the ‘Gene regulatory and signaling networks reconstruction’ section) using the 910 genes selected on the basis of EMT signatures (see the ‘Experimental datasets’ section). The specificity of this SN reconstruction process is demonstrated through a comparison of pyBRAvo with a similar state-of-the-art tool, PyPath (10). We focus on global characteristics of the reconstruction process, such as the size of the retrieved graphs and the computational time. We also deepen this analysis by using classical graph metrics, such as the number of cliques and the nature of the motifs in the retrieved graphs.

#### Recursive upstream retrieval algorithm using PyPath

PyPath is a Python module conceived to query and manipulate the OmniPath database. PyPath provides various functionalities such as the search for protein identifiers and their up- or downstream elements documented in the OmniPath database. PyPath allows searching for direct predecessors from a list of target nodes; however, the complete reconstruction of a SN related to the upstream events from a list of target nodes is not available. Therefore, we implemented an algorithm using the PyPath functions as described in Supplementary Figure S6. This algorithm does not require specific ID management (aliases, synonyms) because OmniPath already provides pre-processed IDs. Indeed, in OmniPath, all identifiers from the original databases (Gene Symbol, Entez Gene ID, Ensembl Gene or Protein, HGNC name, etc.) have been converted to SwissProt. Our tests using this tool were conducted during October 2019.

##### Experimental setup.

To give an overview of the performance of pyBRAvo and PyPath, we evaluated each tool with various settings as shown in Table 2. The evaluation of both tools was based on the following metrics: (1) the reconstruction execution time, (2) the number of nodes and edges of the output graph and (3) the coverage of the input gene list (see the ‘Evaluation metric computation: coverage’ section). For both reconstructions, we chose to recover only signed interactions (pyBRAvo option -unk).

**Table 2.** pyBRAvo allows multiple configurations for the upstream exploration calls as described in the ‘Gene regulatory and signaling networks reconstruction’ section

Tool shortname	Synonyms	Complex	Label	Excluded
	expansion	decomposition	expansion	database
pyBRAvo—fast^a^	No	No	No	Reach
pyBRAvo—synonyms^b^	Yes	No	No	Reach
pyBRAvo—synonyms+complex^c^	Yes	Yes	No	Reach
pyBRAvo—synonyms+complex+label^d^	Yes	Yes	Yes	Reach
PyPath without loading	NA	No	NA	–
PyPath with loading	NA	No	NA	–

^a^Option -fa, ^b^Option -sy, ^c^Option -sy -co, ^d^Option -sy -co -su. The fast option only explores BioPAX display names (bp:displayNames); the *synonyms* option explores synonyms that have been retrieved from the NCBI Gene database; the *complex* option splits a protein complex into its protein components, and these proteins are integrated into the following upstream search and finally, the *label expansion* option allows including several forms (added suffixes, prefixes) of an entity in the upstream search. The PyPath loading option accounts for the time it takes to load the database before the algorithm is executed.

#### Execution time

We compared the execution time (pyBRAvo and PyPath calls where executed from a standard laptop machine equipped with Intel^®^ Xeon^®^ CPU E5-2620 0 @ 2.00GHz x 18 and 32Gb of RAM memory) needed by pyBRAvo and PyPath to separately reconstruct a signed SN from the same dataset, by varying the reconstruction depth from 2 to 10 levels with a step of 2 (see Supplementary Figure S7). This comparison was done using the settings shown in Table 2. We briefly review these results in the following paragraphs, and more details can be found in Supplementary Methods E.

##### Cost of update.

pyBRAvo queries of the private server are faster, although the data may be outdated in the long term. Depending on the call, the private SPARQL endpoint allows pyBRAvo to obtain a reduction of the computation time in a range of 31–49% of the time obtained when querying the PC endpoint. While pyBRAvo allows reconstructing biological networks on ‘fresh’ publicly available data, we note that client–server communication overheads must be considered with respect to computation time. In the context of large-scale biological network reconstructions, we thus recommend deploying a private SPARQL server. This will enhance computation time, as well as limit overloading public resources. A detailed comparison with PyPath computational time for similar queries is shown in Supplementary Methods E.

##### Cost of modeling choice.

The pyBRAvo option *complex decomposition* greatly increases computational time for graph reconstruction (see Supplementary Figure S7). This increase occurs because pyBRAvo includes the protein complexes as nodes in the graph and links them to their protein members. Although PyPath also includes the members of protein complexes, it does not represent a node protein complex in the graph (see Supplementary Methods E). A PyPath modeling choice may generate artificial oscillations between members of a protein complex. In particular, for the reconstruction of 10 levels of depth, the PyPath graph had 6814 interactions; 31.5% of them belonged to a cycle of size 2 (i.e.  a motif $A \rightarrow B$, $B \rightarrow A$). For the pyBRAvo graph, only 5.6% of 5252 interactions were in a cycle of size 2. Moreover, when computing the number of cliques (see Supplementary Figure S8), we observe that the pyBRAvo graph contains at most two cliques of size 5, while PyPath has 1392 of size 5 and its largest clique is of size 10.

##### Cost of identifiers homogenization.

An explicit representation of the PC BioPAX information, using the entity symbols, generates graphs with components that refer to the same entity being artificially disconnected. pyBRAvo proposes to homogenize protein identifiers in two ways (see the ‘Query expansion and network unification’ section): (i) by performing a post-treatment that removes suffixes and prefixes, also called *unification*; and (ii) by performing an upstream exploration of their symbol synonyms and/or performing a label expansion. Therefore, the respective calls (option *synonyms*) are more expensive.

#### Graph

**Figure 3.**
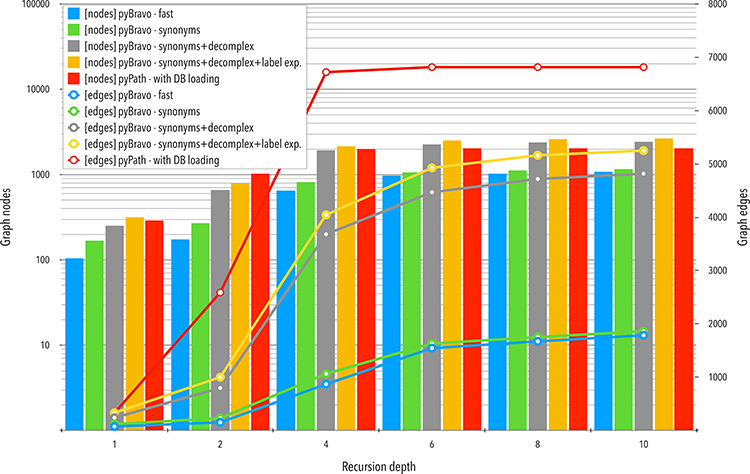
Comparative analyses of the networks obtained with pyBRAvo and PyPath. The horizontal axis represents the level of reconstruction depth of the SN. Bars plot show measures of the number of nodes; lines show the number of edges, the *y*-axis (left) is shown in a logarithmic scale.

The results with respect to graph content are summarized in Figure 3. We do not consider outdated vs.  updated calls of either PyPath or pyBRAvo because the results were identical. We observe that each additional pyBRAvo option, presented above, improves the quantity of nodes and edges of the retrieved graph. This increase in the number of nodes and edges grows rapidly until reaching a plateau at the fourth level of recursion depth. This stabilization likely occurs because further explorations point to components that have already been found. The large gap that opens when using the pyBRAvo—complex call appears because of the new entities created and explored.

We focus now on the signed SNs obtained for the 10-level upstream exploration query with PyPath and pyBRAvo (call pyBRAvo—synonyms+complex+label in Table 2). The PyPath graph is composed of 6814 signed edges, labeled as *stimulation*, *inhibition* and *stimulation and inhibition* (291 edges), while the pyBRAvo graph has 5252 edges labeled as *activation*, *inhibition* and *part_of*. The PyPath graph contains only protein nodes (2049), while the pyBRAvo graph is composed of 2642 nodes of two different kinds, proteins (1571) and complexes (1071). Most of these complexes are protein complexes (1067), while the rest are small molecule complexes. This different nature of the entities may create the observed difference in node and edge size shown in Figure 3.

We measured a coverage of 16.48% of the 910 input genes for the PyPath graph and of 10.9% for the pyBRAvo graph. These results indicate that in both databases (OmniPath and PC), the tools recovered upstream signaling events for only a small fraction of the 910 genes. PyPath information, however, had a better coverage for this dataset. We can see in Table 3 that PyPath and pyBRAvo query different types of resources.

**Table 3.** Sources used to build the graphs using either PyPath (querying OmniPath) or pyBRAvo (querying PC)

Database	Data sources (number of signed interactions)
OmniPath	Adhesome (1347), ARN (707), CA1 (2205), CellPhoneDB (108), Guide2Pharma (88), HPMR (791), Macrophage (1237), NRF2ome (576), PDZBase (156), Ramilowski2015 (575), SignaLink3 (5138), Signor (6466), SPIKE (4947), TRIP (497)
PC	CTD (245), PANTHER (39), PID (2037), Reactome (570)

In Table 4, we show the different topological properties of both graphs. We can observe, for example, that the PyPath graph has more connected components than the pyBRAvo graph (37 vs. 18), suggesting that the information in PC, as interpreted by pyBRAvo, appears easier to assemble with respect to signed controlled signaling reactions. The pyBRAvo graph presents signed self-loops, which are real biological mechanisms useful in dynamic modeling. The PyPath graph shows 15.7% of multiple edges as related to the same pair of nodes, while the proportion is 3.7% in the pyBRAvo graph. This outcome shows that an interaction can be retrieved multiple times by different data sources contained in either Omnipath or PC, and repeated retrieval happens more often in Omnipath than in PC. From the number of cliques computed, we observe that the PyPath graph has a larger proportion of cliques than the pyBRAvo graph (see Supplementary Figure S8). In (25), it was observed that cliques in interaction graphs (protein–protein networks) can be associated with protein complex entities. Since the PyPath graph does not contain entities related to protein complexes, this information may need to be retrieved from an enumeration of cliques; whereas, in the pyBRAvo graph, this enumeration of cliques is not necessary because the graph is composed of protein complex nodes. SNs are known to present a structure in which chains of cascades are combined. From the diameter and characteristic path length information, we observe that the pyBRAvo graph is composed of longer (27 vs. 14 in diameter) chains (directed paths) than PyPath. We interpret these longer chains of entities as being a result of the complex formation in pyBRAvo’s representation. In Supplementary Figure S9, we observe that this distribution of shortest length paths is more dispersed in the pyBRAvo graph compared with the PyPath graph. These chains are an essential way of representing the signaling knowledge; a computational model that includes them enables a better representation of biological reality.

**Table 4.** Network analysis of the PyPath and pyBRAvo signed and directed graphs

Property	PyPath	PyPath^*L*^	pyBRAvo	pyBRAvo^*L*^
N. Nodes	2049	1955	2642	2587
N. Edges	6814	6469	5252	5206
Clustering coefficient	0.066	0.066	0.063	0.026
Connected components	37	1	18	1
Network diameter	14	14	27	27
Percentage of shortest paths	33%	35%	30%	31%
Characteristic path length	4.8	4.9	8.5	8.5
Avg. number of neighbors	5.6	5.5	3.8	3.8
Self-loops	0	0	16	16
Multi-edge node pairs	1076	1000	196	191
Maximal clique size	10	10	5	5
Cliques of size 3	2581	2581	604	604

The upper ‘L’ refers to the largest connected component found in each respective graph. Both graphs represent signaling knowledge as stored in Omnipath and PC, respectively, on October 2019.

**Figure 4.**
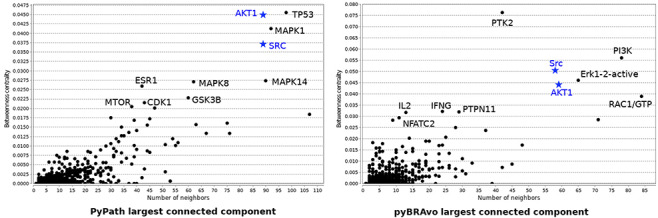
Betweenness centrality vs. number of neighbor nodes for the largest component graph obtained with PyPath and pyBRAvo, respectively, when querying signaling knowledge in Omnipath and PC. The names of the 10 species having the highest betweenness centrality score are shown, and blue names and blue stars correspond to species top-ranked in both graphs.

We also compared the largest connected component of both tools in terms of the betweenness centrality score with respect to the number of neighbors, and plotted the names of the 10 top-ranked species having the highest betweenness centrality score (see Figure 4). We observe that 2 of the 10 top-ranked species are shared in both graphs; whereas, the other species are specific to each approach. A last analysis of these graphs was for detecting the types of motifs within them. To this end, we used the MotifNet web service (26). We observe that several of the 3-size motifs were shared (four out of five for pyBRAvo, and four out of six for PyPath) between both graphs. However, pyBRAvo has fewer motifs including cycles than PyPath (see Supplementary Figure S10). For the case of 4-size motifs (data not shown), the difference is more marked: the PyPath graph contains 69 significant motifs, while the pyBRAvo graph has only 42. The main difference between the types of motifs is the absence of 4-size motifs containing three to six cycles in pyBRAvo. These motif analysis results point to the strong inter-connectivity within the PyPath graph. All in all, these results illustrate how signaling knowledge is currently represented by the different knowledge sources.

The Cytoscape session containing the 10-level SN extracted using pyBRAvo is available online (https://github.com/pyBRAvo/pyBRAvo/blob/master/cytoscape-vis/signaling_10levels_withoutUNK.cys). In Supplementary Figure S11, we visualize two small subgraphs of the predecessors of matrix metallopeptidase 2 (MMP2) protein as generated by pyBRAvo using the *influence graph* and *hypergraph/reaction* options (see Supplementary Methods A.4). This hypergraph/reaction option is specific to pyBRAvo and is not available in PyPath.

### GRNs for drug screening applications

In this section, we use the 39 genes selected on the basis of the metabolic model (see the ‘Experimental datasets’ section) and show how pyBRAvo enables increasing the number of candidate drugs based on a list of target genes through inclusion of genes with a putative non-direct effect on metabolism.

#### Retrieving potential drugs

Based on a list of 40 input genes identified with their gene symbol, we leverage the DrugBank (27) dataset publicly available as linked data through the Bio2RDF (28) SPARQL endpoint (https://bio2rdf.org/sparql). For each of the candidate gene targets, the principle consists of generating a graph pattern that links drugs to gene names through the DrugBank *target* and *gene-name* edges. These patterns are implemented in a SPARQL query that is executed online through the Bio2RDF endpoint. In this query, we filter only drugs with an “approved” status. These 39 gene targets lead to one approved drug.

#### Augmenting drug targets

pyBRAvo is then launched to assemble a GRN with all query expansion features (suffixes, synonyms, complex decomposition) with a single recursion depth and excluding the PC miRTarBase and MSigDB data sources. From the resulting regulation graph, we retrieve all associated activators or inhibitors, which leads to 38 additional candidate target genes.

#### Retrieving potential new drugs

We next launch the DrugBank exploration process again, as described in the previous paragraph, focusing on this new gene list. As the result, we obtain a list of 46 candidate drugs targeting the regulators of the initial gene list. Table 5 summarizes the proposed drug-screening process.

**Table 5.** In the context of *in silico* drug screening, pyBRAvo allows augmenting the list of candidate drugs based on reconstructed gene regulation networks

Initial target genes	Candidate drugs	New target genes	New candidate drugs
39	**1**	38	**46**

We also ran this experiment on the complete list of 2881 genes involved in the direct regulation of metabolic reactions described in the iLivercancer1715 model. This led to 525 approved drugs, a result that is clearly humanly impossible to assess and possibly irrelevant. This result mainly arose from not focusing on the targeted metabolic objective of the network (biomass production). On the other hand, if we restricted the study to the filtered list of 39 genes, we obtained only one drug, which would clearly be unusable in the context of a drug-screening application.

In this experiment, we showed that running pyBRAvo provides a list of candidate drugs with an indirect effect on the biomass production. Although an evaluation of the biological or clinical relevance of the retrieved potential drugs was beyond the scope of this work, we provide the indication of each of the candidate drugs in the Jupyter notebook available online (https://github.com/pyBRAvo/pyBRAvo/blob/master/drugbank-usecase/Notebook/drugbank-metabolism.ipynb). We believe that GRNs and associated metrics such as centrality could provide interesting perspectives when prioritizing drugs in *in silico* screening applications. This experiment can be reproduced based on the online Jupyter notebook.

### System implementation

pyBRAvo was designed as a Python module to be used through an application programming interface in any Python script or directly in Command Line Interfaces. The proposed use cases were aimed at facilitating integration and fine-tuning of pyBRAvo in bioinformatics tools and workflows. pyBRAvo is also provided with several Jupyter notebooks to reproduce the experiments presented here as well as for training purposes. pyBRAvo source code and notebooks are available at https://github.com/pyBRAvo/pyBRAvo.

## Discussion and conclusion

The pyBRAvo framework allows the automatic reconstruction of GRNs and SNs. The reconstruction is based on Semantic Web querying techniques of semantically integrated data sources. A primary novelty relies on the global nature of the obtained networks, and it is based on the iterative reconstruction process that queries knowledge bases combining interactions across all documented pathways.

In pyBRAvo, we choose to use entity names (gene name, protein names, and so forth) to query the data sources for upstream interactions. Another possibility would be to use gene or protein IDs. Our choice was motivated by the multi-scale nature of the regulation and signaling processes. For instance, the transcription of a gene to mRNA and the translation of mRNA to a protein are implicit interactions that are usually not represented in the data sources. It is thus relevant to link a gene with its mRNA and the associated protein to obtain a complete reconstruction when querying the data sources. Furthermore, the reconstruction benefits from several improvements, namely, complex decomposition, label expansions, synonym expansions and network unification, that allow a deeper exploration of the knowledge base. It proves that the use of entity names together with these improvements enables going far beyond the simple use of gene or protein identifiers as shown in Figure 3.

In principle, pyBRAvo shares some characteristics with pyPath, a method that relies on the OmniPath database to reconstruct SNs on a large scale. In a comparison at a coarse-grained scale, the two sets of results appear comparable with respect to the size and the number of edges. Nevertheless, a deeper inspection of the results shows that several artifacts appear in the pyPath reconstruction, such as the large number of cycles, cliques and 4- and 3-size motifs, possibly resulting from protein complexes management in Omnipath (see Table 4). It is highly necessary to reduce such artifact edges when the obtained networks are used as input to build predictive models in both logical and probabilistic frameworks. We observe that PyPath extracted graphs tend to increase the graph connectivity. This signaling flow, carefully detailed in the BioPAX content, is kept when using pyBRAvo to extract the graph.

In addition, these predictive models have to be considered very carefully. Indeed, most of the knowledge aggregated in the databases results from independent experiments. It is crucial to introduce a validation step by testing the model with experimental data, for instance, before using it in predictive approaches. Nevertheless, we strongly believe that adding such an automatic reconstruction step prior to the prediction step will permit more rapid design of large-scale predictive models while limiting errors due to manual data curation tasks.

By leveraging Semantic Web technologies and the BioPAX ontology, pyBRAvo is able to mine any biological network knowledge expressed with commonly accepted pathway terms, concepts and relations. It could additionally be enriched using other external RDF datasets, such as DrugBank or DisGenet (29), with an adapted querying process. This can largely enrich the construction of systems biology models based on disease descriptions for instance. In addition, Semantic Web technologies enable performing complex queries on multiple data sources without the need to locally host possibly massive datasets.

Assembling the vast amount of knowledge that is now publicly available is challenging. pyBRAvo has been designed with a particular focus on data and knowledge reuse. Through this work, we wish to encourage the Systems Biology community to combine their efforts toward developing machine actionable and causality-oriented biological resources, leveraging community standards, and ensuring rich and shared semantics.

## Supplementary Material

baaa113_Supp
