# Supplementary material for: Large-scale regulatory and signaling network assembly through linked open data
Source: Database (Oxford). 2021 Jan 18;2021:baaa113. doi: 10.1093/database/baaa113 (PMC7812716; doi:10.1093/database/baaa113)
Supplement: baaa113_Supp [file baaa113_supp.zip › supMat.pdf]

Large-scale regulatory and signaling network assembly through linked open data — Supplementary Material

Marie Lefebvre, Alban Gaignard, Maxime Folschette, Jérémie Bourdon and Carito Guziolowski

This file contains supplementary Material & Methods, Supplementary Figures and Supplementary Tables related to the article entitled *Large-scale regulatory and signaling network assembly through linked open data.*

Contents

A pyBRAvo method description **2**

B Comparison of PyPath and pyBRAvo **5**

C Comparison of regulatory and signaling networks extracted with pyBRAvo using PC11 and PC12 **5**

D Iggy predictions analysis **5**

E PyBRAvo and PyPath execution time comparison **7**

F Biochemical reactions in Pathway Commons **9**

G Converting the output of pyBRAvo in a Cytoscape graph **9**

List of Figures

S1 A typical gene regulatory network represented in BioPAX 2
S2 Part of a signaling network, represented in BioPAX 3
S3 Basic graph pattern aimed at matching regulation patterns 3
S4 Basic graph pattern aimed at matching signaling pattern 4
S5 Signaling networks retrieved from Pathway Commons information 4
S6 Proposed algorithm to use PyPath to reconstruct a signaling network from the up-stream events of a list of target genes, given as input 5
S7 Evolution of the real CPU time for the reconstruction of a signaling network with regard to the different settings of pyBRAvo and PyPath 8
S8 Distribution of the number of cliques found in the PyPath and pyBRAvo graphs . 9
S9 Distribution of the shortest path length in the PyPath and pyBRAvo graphs . . . 9
S10 Motifs of size 3 for PyPath and pyBRAvo graphs 11
S11 Subgraphs of the 10-level signaling network extracted with pyBRAvo up-stream MMP2 12

List of Tables

S1 Comparison of PC11 and PC12 regulatory network characteristics 6
S2 Comparison of PC11 and PC12 signaling network characteristics 6
S3 Iggy predictions analysis 7
S4 Gene regulatory network extraction using pyBRAvo and its computational model . 7
S5 Sources with biochemical reactions present in Pathway Commons by October 2019 10

A pyBRAvo method description

1 Gene regulatory and signaling networks representation

BioPAX (2) is a community effort aimed at standardizing the representation of biological pathways. Biological pathways are complex objects involving possibly chained biochemical reactions, with specific roles. As a formal ontology, BioPAX precisely defines the concepts (ontology *classes*) and relations (ontology *properties*) underlying complex biological pathway objects and allows to uniformly represent gene regulatory networks and signaling networks.

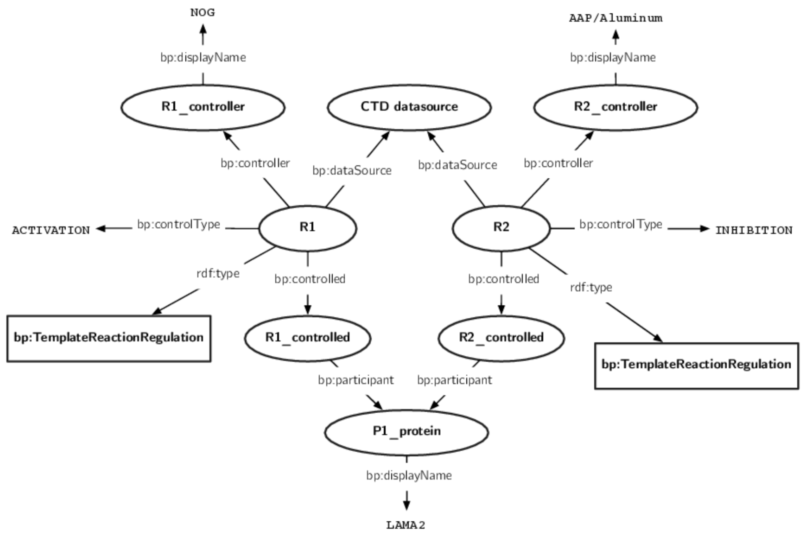

Figure S1: **A typical gene regulatory network represented in BioPAX.**

Figure S1 provides an example of a minimal gene regulatory network (GRN). This GRN shows two biochemical reactions $R1$ and $R2$ having a regulatory role. This is stated for `R1` with the predicate `rdf:type` towards `bp:TemplateReactionRegulation`, meaning that $R1$ has a formal type `TemplateReactionRegulation` as defined in BioPAX. This regulation has a source (`bp:controller`), a target (`bp:controlled`) and a regulatory role (`bp:controlType`). This GRN could be interpreted in natural language with the following statement: *"NOG is an activator of LAMA2; AAP/Aluminum is an inhibitor of LAMA2"*. Although such GRN representations seem rather complex in BioPAX, each concept and relation is made explicit and allows for automated querying and computational navigation. pyBRAvo leverages these terms to automatically assemble gene regulatory networks as well as signaling networks.

Figure S2 provides a slightly different example where the `ELK1` protein catalyses a biochemical reaction consuming and producing the `FOS` protein.

2 Pattern matching expressions

2.1 Regulation pattern matching

The principle consists in searching for all `control` interactions that can be possibly typed as `ACTIVATION` or `INHIBITION`. For each of these interactions we identify who are the `controller` and the `controlled` entities. Controlled entities are represented as `TemplateReactionRegulation` in BioPAX which is an abstraction to represent transcription and translation phenomena for instance. To identify the controlled genes or proteins, we finally search for the `participants` involved in these abstract reactions. See Figure S1 for a visualization of the structure of the regulatory information.

This regulation pattern can be formalized as a basic graph pattern reported in Figure S3, in which the lines represent triplets { `source_node` ; `relation` ; `target_node` }, with variable nodes prefixed with a question mark (in blue).

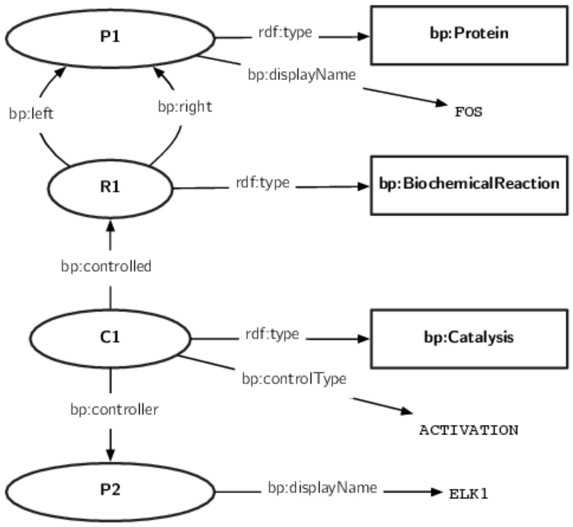

Figure S2: **Part of a signaling network, represented in BioPAX.**

Regulation graph pattern

```
?regulation  is-a  TemplateReactionRegulation .
?regulation  controlType ?controlType .
?regulation  controlled ?regulation_target .
?regulation  controller ?regulation_source .

?regulation_target  participant ?regulated_entity .

?regulation_source  displayName ?controller_name .
?regulated_entity  displayName ?regulated_name .
```

Figure S3: **Basic graph pattern aimed at matching regulation patterns**, i.e., biological entities activating or inhibiting genes.

2.2 Signaling pattern matching

The BioPAX representation of signaling mechanisms differ from regulations since they involve both biochemical and catalysis reactions. In Figure S2 we illustrate the structure of the signaling information. To identify a controlled biological entity, the first step consists in determining all registered biochemical reactions producing it. This is done by retrieving the reactions described with this entity as `right` part of the reaction. Then, once reactions are identified, the second step consists in retrieving all catalysis reactions controlling these biochemical reactions (`bp:controlled` predicate). For each of them, we retrieve the biological entity controlling the catalysis (`bp:controller` predicate). Finally we retrieve its display name and if it exists (`OPTIONAL`) the type of control realized (`bp:controlled` predicate), for instance activation or inhibition. This process can be formalized through the basic graph pattern reported in Figure S4.

3 Output influence graph or hypergraph

PyBRAvo will generate two types of structures to represent the regulatory and signaling knowledge in Pathway Commons (PC): *influence graphs* and *hypergraphs*. These structures will help the further modeling of PC retrieved information, via pyBRAvo, by using different computational modelling approaches.

Influence graphs. An influence graph $G(V, E, \sigma)$ is a signed and directed graph, where V represents the set of nodes, E the set of edges, and $\sigma : E \rightarrow \{+, -, \circ, \emptyset\}$ a labeling representing the nature or effect (activation, inhibition, *part_ of*, or unknown) of the edges. If $s \rightarrow t$ is an edge in the influence graph, representing the activation of species t triggered by s, this implies that *the*

Signaling graph pattern

```
?reaction  bp:right  ?right .

?right  bp:displayName  ?rightName .
?right  rdf:type  ?rightType .
?reaction  bp:left  ?left .
?left  bp:displayName ?leftName .

?catalysis  bp:controlled  ?reaction .
?catalysis  bp:controller  ?controller .
?controller  bp:displayName  ?controllerName .
?controller  rdf:type  ?controllerType .
OPTIONAL { ?catalysis  bp:controlType  ?controlType .}
```

Figure S4: **Basic graph pattern aimed at matching signaling pattern**, i.e., biological entities controlling the production of other entities.

increase of species s induces the increase of species t. PyBRAvo will build influence graphs using gene regulatory and signaling knowledge. For the gene regulatory knowledge, all edges of the graph will represent gene transcriptional regulation or protein-complex formation (edges of type $\circ$). For the signaling, the influence graph will represent the flow of the reaction controllers (see Figure S5) or protein-complex formation. Some PC BioPAX files representing gene regulatory and signaling knowledge, provide a `CONTROL_TYPE` label with *activator* or *inhibitor* information and so we can infer '+' or '−' values in the edges. In other cases this information is not present, and thus we represent it as unknown edges ($\emptyset$).

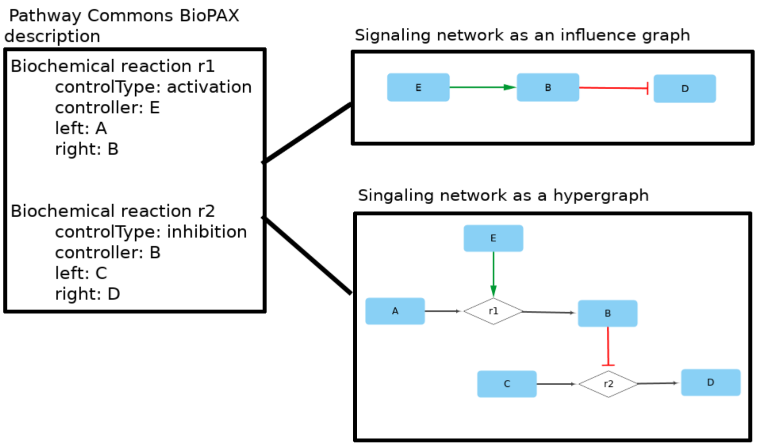

Figure S5: **Signaling networks retrieved from Pathway Commons information.** We illustrate here a simplification of how the signaling knowledge, stored in BioPAX files, is processed by pyBRAvo to retrieve, according to the user's need, two different types of structures: influence graphs and hypergraphs. These structures are extracted as Simple Interaction File (SIF) format, readable by Cytoscape (4), and they can be straightforwardly visualized by following the instructions of Section G.

Hypergraphs. PyBRAvo can also generate from PC signaling knowledge an hypergraph structure. A directed hypergraph $H(V, E)$ consists of two sets V and E. V represents the set of nodes and E, the set of hyperarcs (signaling reactions or complex-formation relations). Each hyperarc $e\langle V_s, t\rangle$ is a pair composed of a non-empty subset V_s of V and a target node $t \in V$. V_s and t appear respectively in the source and target of the hyperarc. Some hyperarcs $r\langle V_s, t\rangle$ represent the reaction controlling the state of node t. The controlling mechanism is represented in the subset V_s ($|V_s| = 2$), that is composed of two elements $V_s = \{c, i\}$ that refer respectively to the controller (c) of the reaction and the input (i) of the reaction, i.e., the species which state will be transformed

because of the controller's effect. This effect is given sometimes with an activation ('+') or inhibition ('−') sign, and in other cases it is unknown (see Figure S5). Other hyperarcs $cplx\langle s, t\rangle$ represent a complex-formation relation, and in that case s represents a protein member of complex t, and this type of relation is labeled as *part_ of*.

B Comparison of PyPath and pyBRAvo

1 Recursive upstream retrieval algorithm using PyPath

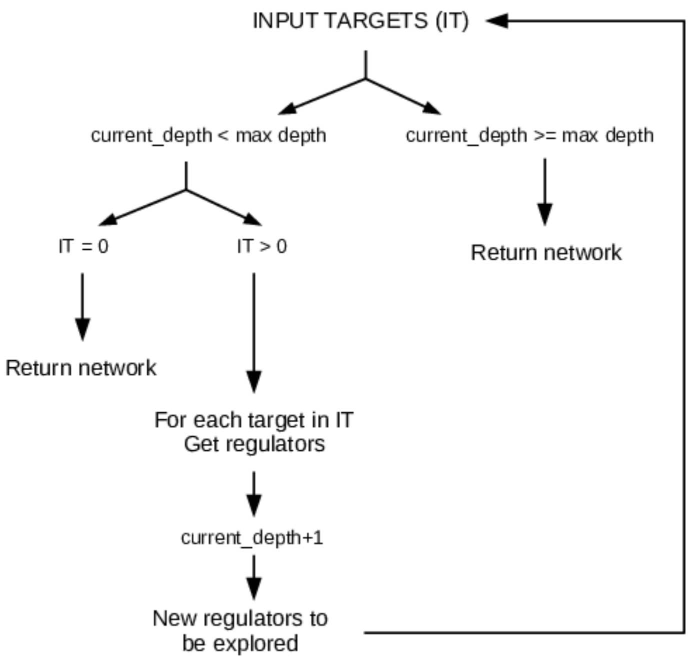

Figure S6: **Proposed algorithm to use PyPath to reconstruct a signaling network from the up-stream events of a list of target genes, given as input.** This algorithm is implemented in a recursive way. The *max_ depth* corresponds to the maximum number of levels to be reconstructed.

C Comparison of regulatory and signaling networks extracted with pyBRAvo using PC11 and PC12

PC was updated on the 24th October 2019 (3) from version 11 to version 12. In Tables S1 and S2 we compare the result of regulation and signaling reconstructions using pyBRAvo on PC11 and PC12, with a depth of 10 and using the same options as in the Results sections 3.1 and 3.2. The results show that the regulation and signaling graphs of PC11 and PC12 are very similar. For regulation, there is a coverage gain of 1.5%, although in one case PC12 removes up to 48 nodes and 142 edges in number. For signaling, there is the same coverage and a maximum of 10 nodes and 7 edges in difference, which may explain the computation time difference.

D Iggy predictions analysis

In Table S3 we enumerate the predictions produced by Iggy in Section 3.1 of the main article. In Table S4 we compare Iggy's predictions matching the experimental data for the cases the graph was built with 2 and 10 exploration levels.

pyBRAvo shortname	Metrics	PC11	PC12
Synonyms+complex+label	Time	98min	80min*
	Unified nodes	1678	1630
	Unified edges	4425	4283
	Coverage (%)	75.9	77.4
Fast+synonyms+complex+label	Time	30min	17min*
	Unified nodes	1619	1617
	Unified edges	4196	4209
	Coverage (%)	75.7	77.4

Table S1: **Comparison of PC11 and PC12 regulatory network characteristics** retrieved using pyBRAvo, for a depth of 10. Note: *These computations were performed on another computer with an Intel Core i7-8565U CPU of 8×1.80GHz, 8Go memory and running Ubuntu 18.04, which may partly explain the difference in the reconstruction time.

pyBRAvo shortname	Metrics	PC11	PC12
Fast	Time	40s	41s
	Unified nodes	1086	1087
	Unified edges	1786	1787
	Coverage (%)	5.05	5.05
Synonyms	Time	1m49s	1m53s
	Unified nodes	1167	1168
	Unified edges	1862	1862
	Coverage (%)	7.25	7.25
Synonyms+complex	Time	11m35s	16m49s
	Unified nodes	2436	2446
	Unified edges	4818	4825
	Coverage (%)	9.12	9.12

Table S2: **Comparison of PC11 and PC12 signaling network characteristics** retrieved using pyBRAvo, for a depth of 10.

**Matching with the experimental data:**	
**Positive predictions:**	TNF CLMP HACD1 SPECC1 GALNT14 CTSK PRKAA2 GUCY1A1 ALKAL2 KAZN MMP23B TENM4 P3H2 LPAR1 P3H3 BICDL2 NFE2L2 ANXA8L1 MEDAG ESR1 TRIM33
**Negative predictions:**	PTEN SPEN MUC1 RPL10 FOXM1 FOXA2 EGR1 DPEP1
**Not matching with the experimental data:**	
**Positive predictions:**	IFNG RALA SRPRA ADRA2A AGXT DNMT3B DAP KLF5 GLMN STK4 ERAL1 PLPPR1 NAT1 FMO4
**Negative predictions:**	TP53BP1 NCOA2 ACKR1 HES1 ARHGEF25 PIK3CA PLPP4 GUCY1B1 APP PNMA8A RFLNA ANOS1 HDGFL3 GALNT16 MBOAT2 GALNT17 SHISAL1 KIRREL1 ESR2 CXCR4
**Not found in experimental data:**	
**Positive predictions:**	Thiosemicarbazones_Copper Disulfiram_Copper Vitallium_analog_titanium_dioxide ormosil_Polyethylene_Glycols NOG TAp63g_tetramer GALNT13 INS dNp63a_tetramer IL4 Estradiol_ESR2
**Negative predictions:**	IFNA1 NSC_689534_Copper IFNA1 pirinixic_acid_PPARA APP_Aluminum ciglitazone_PPARG Polyethyleneimine_Gold

Table S3: **Iggy predictions analysis.** List of the nodes that are predicted using Iggy, classified by their match with the experimental data and their predicted sign (positive or negative).

endpoint	Private	Private	Private	Private
maximum depth (md)	2	2	10	10
actual stop	2	2	5	5
unification	no	yes	no	yes
Time	17min 28s		25min 11s	
Nodes	1550	1474	1792	1678
complexes	243	243	330	330
with small molecules	21	21	27	27
Coverage /910	690	690	691	691
Edges	3405	3380	4455	4425
signed	2833	2808	3749	3719
unsinged	74	74	107	107
PART_OF	449	449	599	599
Predictions	105	56	128	82
+	53	29	70	46
−	47	26	53	33
matching	42	26	54	29
not matching	40	29	52	34
MCOS	149	149	148	148

Table S4: **Gene regulatory network extraction using pyBRAvo and its computational model.** Graph characteristics, computation time, and computational predictions using Iggy for the regulatory networks obtained with pyBRAvo exploring 2 and 10 depth levels. Unified graphs are obtained after merging entity-symbols, therefore they are smaller in number of nodes. MCOS stands for the number of inconsistencies found between graph and dataset. The grey column denotes the results explained in detail in Section 3.1 of the main article.

E PyBRAvo and PyPath execution time comparison

On Figure S7 we show the comparison of the execution time for signaling network reconstruction between PyPath and pyBRAvo for the different exploration levels and different pyBRAvo options described on Section 3.2.1 of the main paper. This comparison allows us to understand different choices/costs of the implementation in the pyBRAvo tool with respect to PyPath; they are explained in detail below and concern: the cost of update, of modeling assumptions, and of data homogenization.

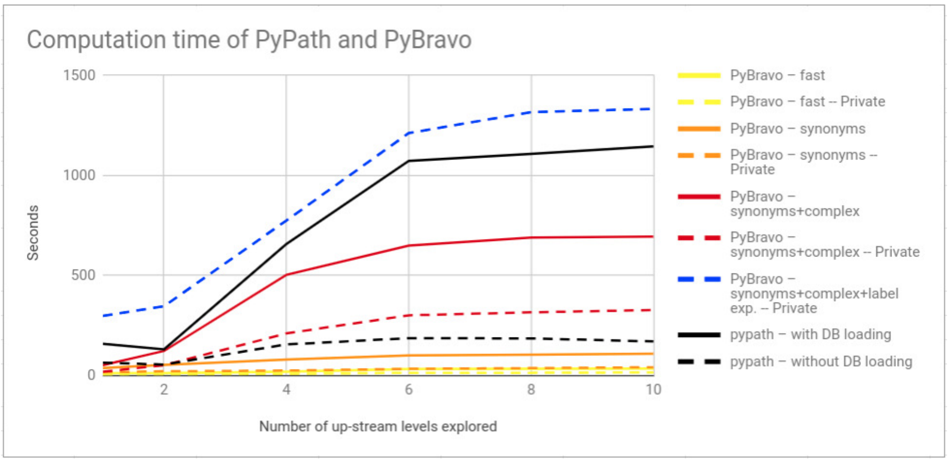

Figure S7: **Evolution of the real CPU time for the reconstruction of a signaling network with regard to the different settings of pyBRAvo and PyPath.** The horizontal axis represents the level of reconstruction depth of the signaling network. The vertical axis represents the execution time in seconds. Dotted lines refer to pyBRAvo computations that used a private SPARQL endpoint and to PyPath computations without loading the OmniPath database.

Cost of update This cost is represented in Figure S7 by the gap between the full and the dashed lines for each analysis. The black line considers the additional cost of loading the last version of the Omnipath database, while the dashed black line considers only the network reconstruction. For the version of October 2019, storing the full Omnipath database required 692MB of storage. With pyBRAvo, we consider two different experimental setups. While plain lines represent access to the public PathwayCommons SPARQL endpoint over the internet, dashed lines represent access to a private SPARQL endpoint deployed locally and populated with a dump of the PathwayCommons database. Compared to PyPath with full database loading, pyBRAvo calls have less computation cost, except for the most complex call, which includes the option of handling synonyms, labels expansion, dissociation of protein-complexes. With respect to PyPath without database loading, the pyBRAvo calls that are faster are `pyBRAvo - synonyms` and `pyBRAvo - fast` (see Table 2). We observe that the call `pyBRAvo - synonyms+complex` (private endpoint) takes slightly more computation time than PyPath (without full database loading) that goes in a range from 1 to 2.6 minutes.

Cost of modeling choice This cost is seen in the red vs. orange curve comparison of Figure S7. In fact, the networks retrieved with the PyPath tool contain only protein nodes, while those retrieved with pyBRAvo contain also protein-complexes. This different representation is related to the choice of pyBRAvo to model the protein-complexes differently than other tools, such as PyPath, PCViz, or ReactomeFIViz. From our point of view, pyBRAvo graphs' causality is more in agreement with mechanistic modeling approaches in which a protein-complex member *is a cause of* the formation of the protein-complex, which itself is the cause of triggering other events. A protein-complex member, in its turn, can also be activated by different signaling pathways. PyBRAvo represents such interactions using the label `PART_OF`. The PyPath tool will also generate a relation between the protein-complex members following Pathway Commons SIF (Simple Interaction Format) conversion rules (1), however these relations only link the protein-complex members and there is no node created to represent the protein-complex entity. This modeling choice may generate artificial oscillations between the protein-complex members.

Cost of identifiers homogenization This cost can be seen in the orange vs. yellow and blue vs. red curves comparison in Figure S7. This cost is needed to handle the different entities' symbols unification in Pathway Commons. The `PyBRAvo - synonyms` (see Table 2) generate more up-stream explorations than the `PyBRAvo - fast` call. This is the same for the `pyBRAvo - synonyms+complex+label` with respect to the `pyBRAvo - synonyms+complex` call. Therefore the resulting graphs have an increased size in terms of nodes and edges. This homogenization step is not needed for the PyPath tool since the OmniPath database is better curated

than Pathway Commons in this respect.

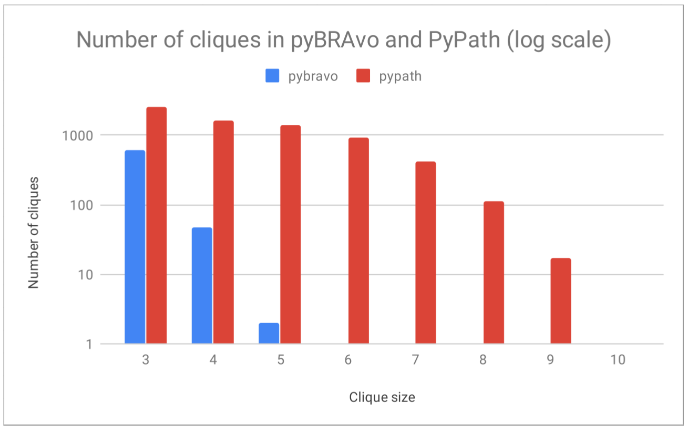

Figure S8: **Distribution of the number of cliques found in the PyPath and pyBRAvo graphs** of Section 3.2 in the main article. The x-axis (in logarithmic scale) represents the clique size, the y-axis the number of cliques found for each size. In the pyBRAvo graph there were 2 cliques of size 5 and 0 of size 6. In the PyPath graph there was 1 clique of size 10.

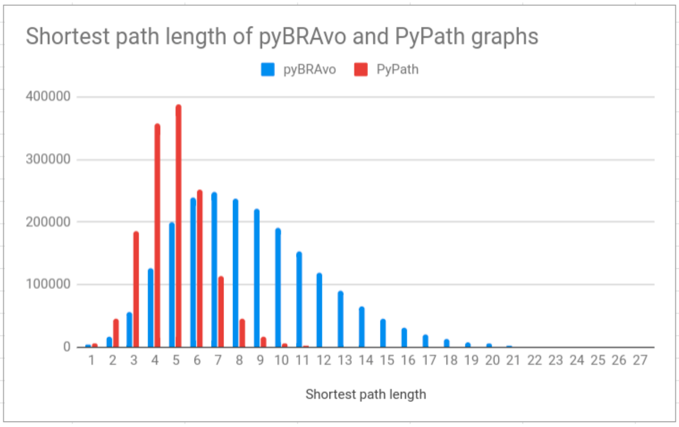

Figure S9: **Distribution of the shortest path length in the PyPath and pyBRAvo graphs** of Section 3.2 in the main article. The x-axis represents the shortest path size between any two elements, the y-axis the number of shortest paths of this size.

F Biochemical reactions in Pathway Commons

In Table S5 we show the number of biochemical reactions present in Pathway Commons, distributed across the different resources present in this database. To obtain these results we queried the RDF endpoint of Pathway Commons on October 2019.

G Converting the output of pyBRAvo in a Cytoscape graph

Running pyBRAvo typically generates two SIF (Simple Interaction Format) files named by default: (1) `out-unified.sif` and (2) `out.sif`. These files correspond to the unified and non-unified versions of the graph as explained in Section 2.2.2 of the main article (Network unification). A user can import one of these SIF files into Cytoscape with the menu: `File / Import / Network from File`. This allows the user to visualize the structure of the graph contained in the SIF file.

Information can also be attached to the nodes and edges of a Cytoscape graph. For instance, along with the generated SIF files, pyBRAvo also outputs two files named by default: (1) `out-unified-provenance.csv` and (2) `out-provenance.csv` that correspond to the database

Source	Number of biochemical reactions
Reach	1025511
Reactome	13834
PhosphoSite	12079
DrugBank	4773
Recon X	4266
PANTHER	3868
CTD	3225
pid	2864
KEGG	2826
NetPath	2014
HumanCyc	1786
INOH	1586
WikiPathways	15

Table S5: **Sources with biochemical reactions present in Pathway Commons by October 2019.**

provenance of the edges contained in the unified and non-unified SIF files. Thus, after importing one of the two SIF files, a user can also import the corresponding provenance file by using menu entry: `File / Import / Table from File` and choosing `Edge Table Columns` in the dialog window. After that, each edge will have its provenance information attached, which can be viewed in the `Edges` tab of the bottom Table Panel in Cytoscape. The same procedure also allows to import information on nodes by selecting a relevant file and choosing `Node Table Columns` in the dialog window. This can be useful for differential expression data (`icgc.syn.csv`) or Iggy computation results (`nodes-iggy.csv`); note that the field separator is the tabulation for these files, which must be specified accordingly in the dialog window.

Finally, different styles (edge tips, color, label...) can be applied to edges and nodes depending on the information attached (type of regulation, provenance, over/under-expression of genes...). This can be done manually by using the `Style` tab of the left Control Panel. A pre-made style is also available in the sources as file `bravo-style.xml` in order to obtain the same result as the graph figures in this Supplementary Material or as in Figure 2 of the main article. It can be imported with `File / Import / Style from File` and selected under the `Style` tab by choosing the style `BRAvo`.

		PyPath		pyBRAvo		Type
		Instances	Z-score	Instances	Z-score	
1	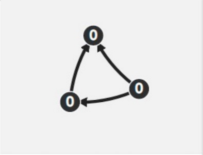	648	5.7	487	28.3	Feed Forward Loop
2	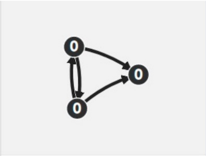	273	15	27	15.2	Sustained Input Switch
3	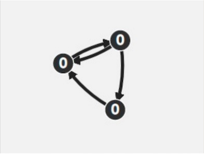	63	1.8	21	11.5	
4	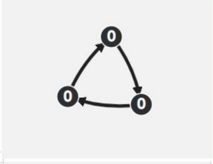			50	10.4	Feed Back Loop
5	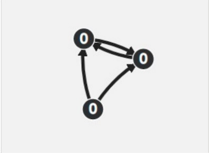	698	79.4	22	13.4	
6	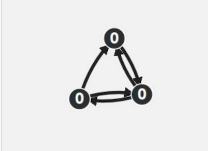	302	10.3			Mixed Feed Back Loop
7	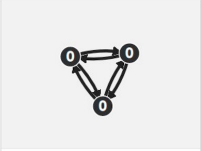	597	193.8			

Figure S10: **Motifs of size 3 for PyPath and pyBRAvo graphs.** We list the more significant motifs obtained in both graphs as well as their type. Red boxes refer to unmatched information, and green, to shared motifs. The motif images and significance were obtained using the web-service MotifNet (5). Our analyses were ran using 1000 random networks for the Z-score computation and fixing as maximal P-value 0.005. Green boxes refer to common motifs (between pyBRAvo and PyPath) while red, to not found motifs.

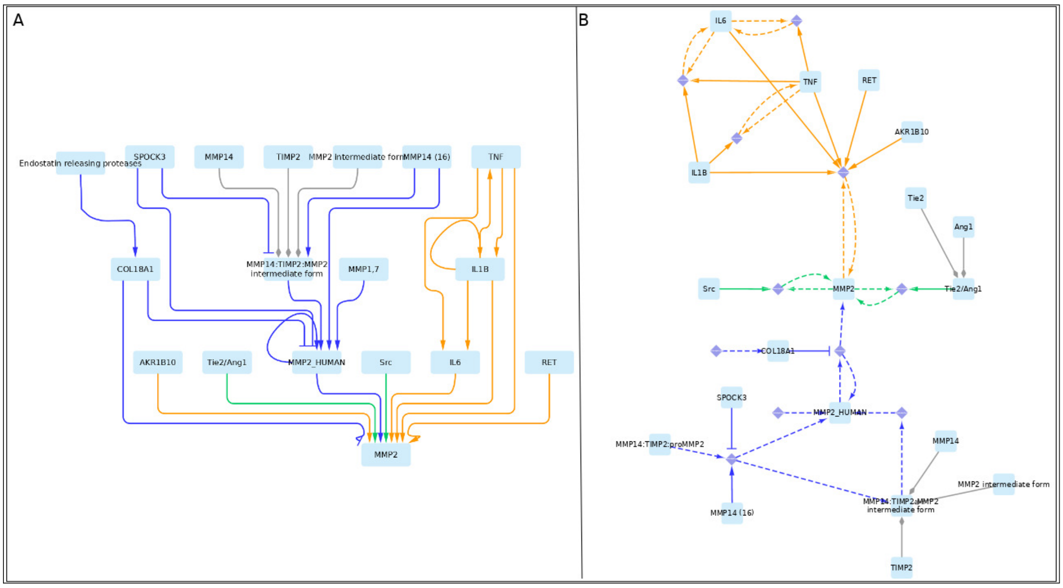

Figure S11: **Subgraphs of the 10-level signaling network extracted with pyBRAvo upstream MMP2.** The color code of the edges gives their provenance: orange for CTD, green for PID, blue for Reactome, and gray for the expansion of protein-complexes. The edge tips depend on the interactions type: triangle for an activation, bar for an inhibition, and diamond for a protein-complex association. **(A)** shows the influence graph; **(B)**, the reaction graph. Reactions are represented by diamond nodes and the left and right sides of the reaction are represented by dashed lines. This graphical export was made with Cytoscape 3.7.0 (4) according to the instructions of Section G.

References

1. Cerami, E. G., Gross, B. E., Demir, E., Rodchenkov, I., Babur, O., Anwar, N., Schultz, N., Bader, G. D., and Sander, C. (2011). Pathway Commons, a web resource for biological pathway data. *Nucleic Acids Res.*, 39:D685–690.

2. Demir, E., Cary, M. P., Paley, S., Fukuda, K., Lemer, C., Vastrik, I., Wu, G., D'Eustachio, P., Schaefer, C., Luciano, J., Schacherer, F., Martinez-Flores, I., Hu, Z., Jimenez-Jacinto, V., Joshi-Tope, G., Kandasamy, K., Lopez-Fuentes, A. C., Mi, H., Pichler, E., Rodchenkov, I., Splendiani, A., Tkachev, S., Zucker, J., Gopinath, G., Rajasimha, H., Ramakrishnan, R., Shah, I., Syed, M., Anwar, N., Babur, Ö., Blinov, M., Brauner, E., Corwin, D., Donaldson, S., Gibbons, F., Goldberg, R., Hornbeck, P., Luna, A., Murray-Rust, P., Neumann, E., Ruebenacker, O., Samwald, M., van Iersel, M., Wimalaratne, S., Allen, K., Braun, B., Whirl-Carrillo, M., Cheung, K.-H., Dahlquist, K., Finney, A., Gillespie, M., Glass, E., Gong, L., Haw, R., Honig, M., Hubaut, O., Kane, D., Krupa, S., Kutmon, M., Leonard, J., Marks, D., Merberg, D., Petri, V., Pico, A., Ravenscroft, D., Ren, L., Shah, N., Sunshine, M., Tang, R., Whaley, R., Letovksy, S., Buetow, K. H., Rzhetsky, A., Schachter, V., Sobral, B. S., Dogrusoz, U., McWeeney, S., Aladjem, M., Birney, E., Collado-Vides, J., Goto, S., Hucka, M., Le Novère, N., Maltsev, N., Pandey, A., Thomas, P., Wingender, E., Karp, P. D., Sander, C., and Bader, G. D. (2010). The BioPAX community standard for pathway data sharing. *Nature Biotechnology*, 28:935.

3. Rodchenkov, I., Babur, O., Luna, A., Aksoy, B. A., Wong, J. V., Fong, D., Franz, M., Siper, M. C., Cheung, M., Wrana, M., Mistry, H., Mosier, L., Dlin, J., Wen, Q., O'Callaghan, C., Li, W., Elder, G., Smith, P. T., Dallago, C., Cerami, E., Gross, B., Dogrusoz, U., Demir, E., Bader, G. D., and Sander, C. (2019). Pathway Commons 2019 Update: integration, analysis and exploration of pathway data. *Nucleic Acids Research.* gkz946.

4. Shannon, P., Markiel, A., Ozier, O., Baliga, N. S., Wang, J. T., Ramage, D., Amin, N., Schwikowski, B., and Ideker, T. (2003). Cytoscape: a software environment for integrated models of biomolecular interaction networks. *Genome Res.*, 13(11):2498–2504.

5. Smoly, I. Y., Lerman, E., Ziv-Ukelson, M., and Yeger-Lotem, E. (2017). MotifNet: a web-server for network motif analysis. *Bioinformatics*, 33(12):1907–1909.
